# Scalable Wire‐Type Asymmetric Pseudocapacitor Achieving High Volumetric Energy/Power Densities and Ultralong Cycling Stability of 100 000 Times

**DOI:** 10.1002/advs.201802067

**Published:** 2019-03-07

**Authors:** Qiuyue Gui, Lingxia Wu, Yuanyuan Li, Jinping Liu

**Affiliations:** ^1^ School of Chemistry Chemical Engineering and Life Science and State Key Laboratory of Advanced Technology for Materials Synthesis and Processing Wuhan University of Technology Wuhan Hubei 430070 P. R. China; ^2^ Institute of Nanoscience and Nanotechnology Department of Physics Central China Normal University Wuhan Hubei 430079 P. R. China; ^3^ School of Optical and Electronic Information Huazhong University of Science and Technology Wuhan 430074 P. R. China

**Keywords:** cycling stability, energy/power density, flexible wire asymmetric pseudocapacitors, quasi‐solid state, scale‐up design

## Abstract

Wire‐shaped asymmetric pseudocapacitors with both pseudocapacitive cathode and anode are promising in facilitating device assembly and provide highly efficient power sources for wearable electronics. However, it is a great challenge to simultaneously obtain high energy and power as well as ultralong cycling life for practical demands of such devices. Herein, a device design with new cathode/anode coupling is proposed to achieve excellent comprehensive performance in a wire‐type quasi‐solid‐state asymmetric pseudocapacitor (WQAP). The hierarchical α‐MnO_2_ nanorod@δ‐MnO_2_ nanosheet array cathode and MoO_2_@C nanofilm anode are directly grown on flexible tiny Ti wires by well‐established hydrothermal and electrodeposition techniques, which ensures rapid charge/mass transport kinetics and the sufficient utilization of pseudocapacitance. The nanoarray/film electrode also facilitates integration with gel electrolyte of polyvinyl alcohol–LiCl, guaranteeing the durability. The resulting WQAP with 2.0 V voltage delivers high volumetric energy and power densities (9.53 mWh cm^−3^ and 22720 mW cm^−3^, respectively) as well as outstanding cycling stability over 100 000 times, surpassing all the previously reported WQAPs. In addition, the device can be facilely connected in parallel or in series with minimal internal resistance, and be fabricated at the 1 m scale with excellent flexibility. This work opens the way to develop high‐performance integrated wire supercapacitors.

## 1 Introduction

With the rapid development of multifunctional, smart, wearable, and miniaturized electronics as well as micro‐/nano‐electromechanical systems, searching for new types of matchable energy storage devices is becoming critically important.^1^, ^2^, ^3^, ^4^, ^5^, ^6^, ^7^ Of the various emerging power sources, flexible solid‐state supercapacitors (SCs) have attracted great interests due to their multifaceted characteristics of high power density, long cycle life, high safety, reduced device thickness, and outstanding mechanical flexibility.^8^, ^9^ In particular, wire‐type SC with 1D flexible architecture and micro‐/submicrosized diameter has been proposed in recent years.^10^, ^11^, ^12^, ^13^ This kind of SC exhibits perfect flexibility so that it could even be twisted and stretched.^12^ Moreover, such devices can be engineered into any shape, woven/knitted into large size, and applied in many places without restrictions, which is much suitable for powering future wearable or miniaturized electronics.

To construct a wire‐type SC and improve its energy storage capability in limited space, to date, carbonaceous fibers (microsized carbon, carbon nanotubes, graphene, etc.) and carbon–metal compounds/conducting polymer hybrid fibers have been dominantly used.^3^, ^5^, ^8^, ^9^, ^10^, ^14^, ^15^, ^16^, ^17^, ^18^, ^19^ These electrode fibers were facilely synthesized with strong strength and good electrical conductivity. Due to the easy device assembly, a symmetric cell structure was initially chosen. However, the output voltage was generally smaller than 1 V, greatly limiting the volumetric energy density of the SC device.^9^, ^10^, ^11^, ^12^, ^13^, ^14^, ^15^ To increase the device energy density, obtaining high cell capacitance (*C*) and voltage (*V*) is highly necessary on the basis of the equation *E* = 1/2*CV* ^2^.^8^, ^20^ Therefore, utilizing high‐capacitance pseudocapacitive electrode materials to construct asymmetric cell should be one of the best choices. Nevertheless, it is not easy to assemble effective asymmetric wire‐type SC by simply paring two fibers if only one fiber is pseudocapacitive and the other one is electric double layer type. This is because of the difficulty in achieving the charge balance (*Q*_+_ and *Q*_−_) with similar fiber length and volume. Attempts have been made to construct asymmetric structure by twisting a longer carbonaceous electrode fiber around a shorter straight pseudocapacitive fiber.^21^ Despite effectiveness, the device volume was increased accordingly. The development of advanced asymmetric wire‐type SCs is still quite challenging.

Currently, one of the alternative choices is to develop wire‐type SCs using two pseudocapacitive electrodes (metal oxides/nitrides, conducting polymers, etc.).^22^ In conventional SCs, manganese dioxide (MnO_2_) is the most popular pseudocapacitive positive electrode material due to high theoretical specific capacitance, natural abundance, low cost, and low toxicity,^23^ while molybdenum dioxide (MoO_2_) is a potential pseudocapacitive negative electrode material^24^, ^25^, ^26^, ^27^ beneficial from its multiple valent states and metallic conductivity at room temperature (>1 × 10^4^ S cm^−1^).^28^, ^29^ Nevertheless, wire‐type SCs assembled from MnO_2_ and MoO_2_ have rarely been reported, and the energy and power densities of wire devices still need to be much improved. In particular, the cycling stability of pseudocapacitive devices is generally limited to thousands of cycles,^30^ which is far from the demand of practical applications. To address these issues, it is highly necessary to synthesize Mn and Mo oxide electrodes with uniform nano‐/microstructures, rational capacitance control, and robust flexibility retention.

**Figure 1:**
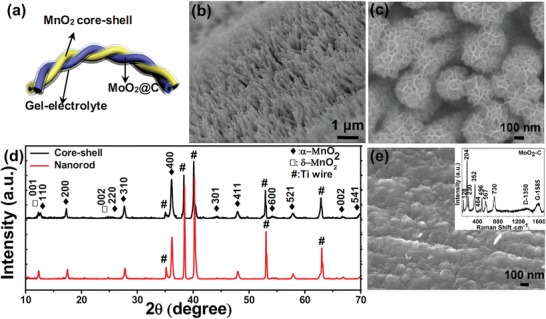
a) Schematic illustration of the WQAP device structure, in which the MnO_2_ core–shell nanorod array positive electrode and the MoO_2_@C nanofilm negative electrode are encapsulated by a thin gel electrolyte of PVA–LiCl and twisted. b,c) SEM images of α‐MnO_2_ nanorod array and hierarchical MnO_2_ core–shell nanoarray on the Ti wire. d) XRD patterns. e) SEM image of MoO_2_@C nanofilm. The inset shows the Raman spectrum.

Herein, we present a novel wire‐shaped quasi‐solid‐state asymmetric pseudocapacitor (WQAP) based on hierarchical biphase core–shell nano‐MnO_2_ cathode and MoO_2_@C continuous nanofilm anode. The two electrodes are designed by directly growing the oxides on 50 µm diameter thin Ti wires via stepped hydrothermal and electrodeposition, respectively. The electrodeposition technique allows the precise control of the nanofilm loading and thus enables facile charge balance between the positive and negative electrodes. Such electrode architectures would ensure fast charge transfer kinetics,^31^, ^32^ robust flexibility, and the intimate interfacial contact with polyvinyl alcohol (PVA)–LiCl quasi‐solid‐state electrolyte. The electrolyte isolates and stabilizes the twisted cathode and anode wires to achieve an integrated WQAP device (**Figure** **1**a), which exhibits a high output voltage of 2.0 V, high volumetric energy and power densities (9.53 mWh cm^−3^ and 22 720 mW cm^−3^, respectively), and ultralong cycling stability over 100 000 times (≈97.36% capacity retention). Our WQAP can also be easily scaled up to 1 m, designed in parallel or in series, and power small electronics efficiently.

## 2 Results and Discussion

### 2.1 Characterizations of the Electrodes

The MnO_2_ cathode was synthesized by a two‐step hydrothermal method. Scanning electron microscope (SEM) images in Figure 1b and Figure S1 in the Supporting Information show that after the first hydrothermal process uniform nanorods have been radially grown on the Ti wire. With the surface carbon coating and subsequent hydrothermal redox reaction with KMnO_4_, hierarchical core/shell nanorod array is produced. As shown in Figure 1c, each original nanorod has been homogeneously deposited with interconnected nanosheets, forming an ordered 3D architecture. The hierarchical nanorods have average diameter of ≈300 nm. Such a highly porous structure is believed to greatly enhance the contact with electrolyte and increase the areal capacitance^7^; in the meantime, the ion diffusion into the inner nanorod core will not be hindered as the outer δ‐MnO_2_ has a layered structure that can provide direct ion transport channels. X‐ray diffraction (XRD) patterns in Figure 1d clearly indicate that the nanorods are α‐MnO_2_ (JCPDS Card No. 44‐141) and the latter grown nanosheets are birnessite‐type δ‐MnO_2_ with the peak at ≈12.3° indexed to (001) plane (JCPDS Card No. 80‐1098). The pure phase of α‐MnO_2_ nanorod and biphase of the hierarchical MnO_2_ can be further evidenced by Raman spectroscopy (Figure S2, Supporting Information). The wire anode was attained by electroreduction of (NH_4_)_6_Mo_7_O_24_ aqueous solution and subsequent oxide deposition (electrodeposition). In order to increase the deposited film's electrical conductivity, glucose was introduced into the electrolyte, which was in situ incorporated into the film and carbonized to conductive carbon during the annealing. Figure 1e reveals the continuous nanofilm morphology of the resulting composite. XRD result in Figure S3 in the Supporting Information indicates that all the diffraction peaks are indexed to a MoO_2_ monoclinic phase (JCPDS Card No. 65‐5787). The presence of carbon is confirmed by Raman spectroscopy (inset in Figure 1e). In the Raman spectrum, the characteristic D and G bands of carbon at 1350 and 1585 cm^−1^ as well as those of nano‐MoO_2_ around 730, 567, 496, 352, and 204 cm^−1^ are well observed.^33^, ^34^

**Figure 2:**
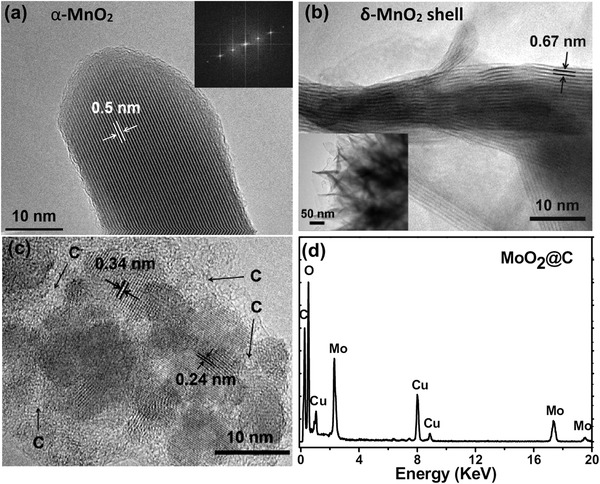
a) HRTEM image of the α‐MnO_2_ nanorod. The inset is the corresponding fast Fourier transform pattern. b) HRTEM image of the δ‐MnO_2_ nanosheet. Bottom‐left inset is the low‐magnification TEM image. c) HRTEM and d) EDX spectra of the MoO_2_@C nanofilm.

The detailed microstructure of the two electrodes was further investigated by transmission electron microscopy (TEM; **Figure** **2**). In particular, interplanar spacings of 0.5 and 0.67 nm are observed for the nanorods and curling nanosheets, corresponding to the (200) plane of α‐MnO_2_ and (001) plane of δ‐MnO_2_ (Figure 2a,b), respectively. This result confirms the intriguing hierarchical biphase architecture of α‐MnO_2_ nanorod@δ‐MnO_2_ nanosheet. High‐resolution TEM (HRTEM) image in Figure 2c uncovers that well‐crystalline MoO_2_ nanoparticles with diameters of 5–10 nm have been uniformly dispersed within continuous carbon matrix. Such a structure will be much beneficial for rapid electron transport and superior electrochemical stability. Lattice distances of 0.34 and 0.24 nm can be detected, corresponding to the (011) and (111) planes of monoclinic MoO_2_. Energy‐dispersive X‐ray spectroscopy (EDX) result in Figure 2d further proves the existence of O, Mo, and C elements in the anode film, in which the Cu signals are from the Cu grid used to load the TEM sample.

### 2.2 Charge Storage Properties of Cathode and Anode

**Figure 3:**
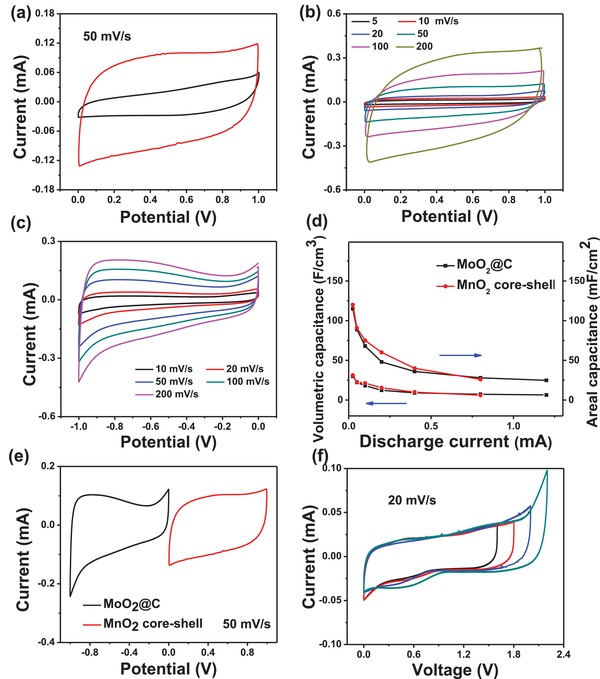
a) CV curves of the MnO_2_ core–shell nanorod array and the α‐MnO_2_ nanorod array. b) CVs of the MnO_2_ core–shell nanorod array cathode at different scan rates. c) CVs of the MoO_2_@C nanofilm anode. d) Rate capability of the cathode and anode. e) Comparative CVs of the cathode and anode. f) CV profiles of the α‐MnO_2_@δ‐MnO_2_//MoO_2_@C full cell at different voltage windows.

Electrochemical performance was first investigated in a three‐electrode system in 2 m LiCl aqueous electrolyte. The cyclic voltammetry (CV) curves of α‐MnO_2_ nanorod array and α‐MnO_2_@δ‐MnO_2_ core–shell array electrodes with the same length are comparatively shown in **Figure** **3**a. With the growth of δ‐MnO_2_ nanosheets, the capacitance of positive electrode is drastically increased more than twice, evidenced by the expanded CV area. This capacitance enhancement is indicative of the merits of hierarchical MnO_2_ architecture that provides abundant active surfaces for pseudocapacitive reaction. CVs at 5–200 mV s^−1^ in Figure 3b always demonstrate quasi‐rectangular shape, reflecting the well pseudocapacitive behavior of MnO_2_ wire cathode. Similar pseudocapacitive performance is also observed for our MoO_2_@C nanofilm wire anode within the negative potential window of −1.0 to 0 V versus Ag/AgCl, as shown in Figure 3c. The charge storage mechanism is considered as the well‐known surface intercalation/deintercalation of Li^+^.^7^, ^35^ Galvanostatic charge–discharge profiles of the two wire electrodes are displayed in Figure S4 in the Supporting Information, both exhibiting relatively linear voltage–time plots at various currents. The rate performance plots of volumetric/areal capacitance versus discharge current are further shown in Figure 3d. It is obvious that within a wide current range, the MnO_2_ core–shell cathode always demonstrates similar capacitance to MoO_2_@C anode, indicative of good charge balance. Both the wire electrodes exhibit excellent rate capability. For instance, at the current of 0.05 mA, the areal and volumetric capacitances of MoO_2_@C anode are estimated as high as 115 mF cm^−2^ and 29.95 F cm^−3^, respectively. With the current increased 24 times to 1.2 mA, ≈21.6% of the initial capacitance can be retained. Electrodeposition of 32 cycles is found to be the optimized condition to achieve the charge balance between the cathode and anode. Based on the CVs in Figure 3e, such two wire electrodes are expected to be assembled into a full‐cell SC with voltage around 2.0 V. This speculation is evidenced in Figure 3f and Figures S5 and S6 in the Supporting Information, in which the CV profiles of the device show quasi‐rectangular shape with the voltage window ≤2.0 V. When the voltage is higher than 2.0 V, the CVs are distorted due to the water electrolysis as typically observed in traditional aqueous batteries and supercapacitors.^36^

### 2.3 Assembly and Electrochemical Performance of WQAP Device

**Figure 4:**
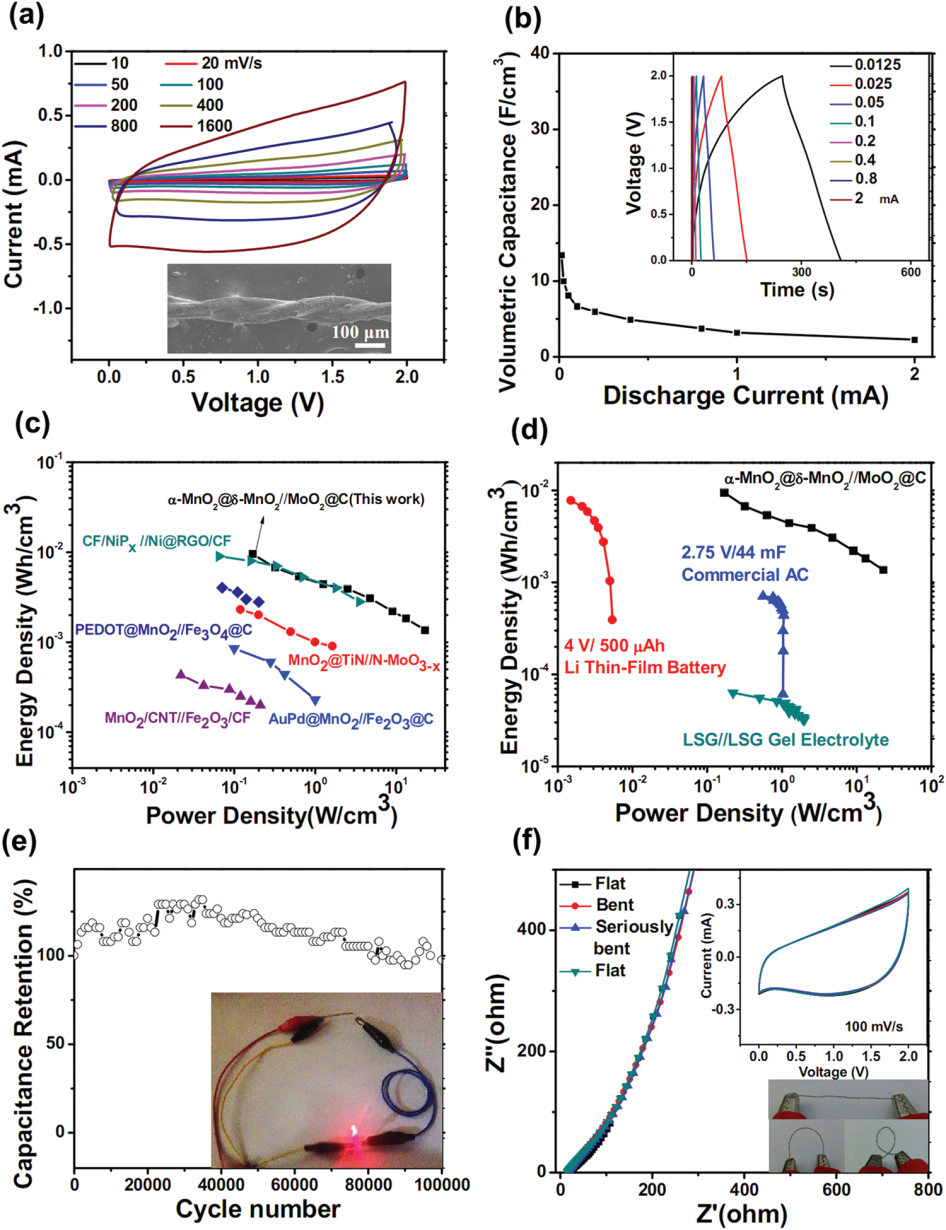
WQAP device: a) CV curves and the SEM device image. b) Rate performance. Inset shows the galvanostatic charge–discharge curves at various currents. Volumetric energy and power densities of the device compared with c) recent reports and d) commercially available state‐of‐the‐art devices. e) Cyclic performance. Inset shows a red LED powered by a single WQAP. f) EIS and the CV curves (inset) of the device at different bending states (inset).

Next, a WQAP device (α‐MnO_2_@δ‐MnO_2_//MoO_2_@C) was developed using the above Mn and Mo oxide–based wire electrodes and PVA–LiCl gel electrolyte, which also serves as the separator. SEM image of the device is given in the inset of **Figure** **4**a, from which an integrated twisted wire structure can be confirmed. Figure 4a also depicts typical CVs of the quasi‐solid‐state device between 0 and 2.0 V at scan rates ranging from 10 to 1600 mV s^−1^. Even at an ultrahigh rate of 1600 mV s^−1^, the CV still retains a relatively rectangular shape without obvious distortion. Galvanostatic charge–discharge curves of the device at different currents are further illustrated in the inset of Figure 4b. The almost linear charging/discharging profiles as well as their symmetry reveal the good capacitive characteristics, in a good agreement with the CV result. Based on these results, the plot of calculated volumetric capacitance versus current is shown in Figure 4b. The rate performance based on areal capacitance is also provided in Figure S7 in the Supporting Information. It can be seen that our device exhibits high volumetric capacitance and areal capacitance of 13.45 F cm^−3^ and 31.7 mF cm^−2^ at 0.0125 mA, respectively. By increasing the current 8 times to 0.1 mA, ≈58.4% of the initial capacitance can be retained; even with the 80 times increase to 1 mA, still ≈26.4% is retained, demonstrating excellent rate performance of the quasi‐solid‐state device.

Power density and energy density are two important parameters to manifest the application potentials of SC devices. Figure 4c,d displays the Ragone plots of volumetric energy density versus power density of our device, in which the previously reported WQAP devices and commercial thin‐film Li battery as well as the state‐of‐the‐art SCs are included for comparison. The detailed parameter comparison of these devices is summarized in **Table** **1**. As can be seen, our quasi‐solid‐state device delivers a high energy density of 9.53 mWh cm^−3^ at a power density of 167.78 mW cm^−3^. Even at the power density as high as 22.72 W cm^−3^, an energy density of 1.37 mWh cm^−3^ is still attained. The maximum volumetric energy density is significantly higher than that of symmetric wire‐shaped SCs^3^, ^12^, ^15^, ^17^, ^37^, ^38^, ^39^ and asymmetric wire SCs^8^, ^40^, ^41^ such as Ni(OH)_2_//C (2.16 mWh cm^−3^ at 38.85 mW cm^−3^)^8^ and CoSe_2_@PPy//AC (2.63 mWh cm^−3^ at 14 mW cm^−3^),^41^ and is better than most of previous WQAP devices based on both pseudocapacitive cathode and anode such as MnO_2_@TiN//N‐MoO_3−_ *_x_* (2.29 mWh cm^−3^ at 120 mW cm^−3^),^27^ MnO_2_/carbon nanotube (CNT)//Fe_2_O_3_/carbon fiber (CF) (0.43 mWh cm^−3^ at 20 mW cm^−3^),[[qv: 22f]] AuPd@MnO_2_//Fe_2_O_3_@C (0.85 mWh cm^−3^ at 100 mW cm^−3^),[[qv: 42a]] poly(3,4‐ethylenedioxythiophene) (PEDOT)@MnO_2_//Fe_3_O_4_@C (4.02 mWh cm^−3^ at 200 mW cm^−3^),[[qv: 42b]] and CF/NiP*_x_*//Ni@reduced graphene oxide (RGO)/CF (8.97 mWh cm^−3^ at 80 mW cm^−3^).[[qv: 42c]] In addition, the volumetric energy densities of our device are comparable to that of commercial 4 V/500 µAh thin‐film lithium battery (0.3–10 mWh cm^−3^)^43^ in Figure 4d, while the volumetric power densities are even slightly superior to the commercially available 2.75 V/44 mF SC^44^ and the laser‐scribed graphene (LSG)//LSG.^45^ Figure 4e shows the cycling performance of our α‐MnO_2_@δ‐MnO_2_//MoO_2_@C device up to 100 000 times. After such a long‐term cycling, 97.36% of the initial capacity can be retained with negligible fading rate. This excellent cycling stability is among the best and has never been demonstrated in WQAP devices (see Table 1 for details; e.g., MnO_2_/CNT//Fe_2_O_3_/CF: 10^4^ cycles, 80% retention[[qv: 22f]]; MnO_2_@TiN//N‐MoO_3−_ *_x_*: 5000 cycles, 80.3% retention^27^). The above results are quite encouraging, which unambiguously reveal the merits of growing pseudocapacitive materials with elaborate micro‐/nanostructures directly on wire current collectors.

**Table 1:** Comparison of electrochemical performance of recently reported WQAPs

Device and materials	*C*_A_ [mF cm^−2^]	*C*_V_ [F cm^−3^]	*E*_V_ [mWh cm^−3^]	*P*_V_ [mW cm^−3^]	Cycle life	Voltage [V]	Ref.
α‐MnO_2_@δ‐MnO_2_//MoO_2_@C	31.7	13.45	9.53	22720	10^5^ (97.36%)	2.0	Our work
MnO_2_/CNT//Fe_2_O_3_/CF	–	0.67	0.43	210	10^4^ (80%)	2.2	[[qv: 22f]]
MnO_2_@TiN//N‐MoO_3−_ *_x_*	–	4.1	2.29	1640	5000 (80.3%)	2.0	27
AuPd@MnO_2_//Fe_2_O_3_@C	–	2.46	0.85	1000	4000 (94%)	1.575	[[qv: 42a]]
PEDOT@MnO_2_//Fe_3_O_4_@C	60	7.23	4.02	360	800 (80%)	2.0	[[qv: 42b]]
CF/NiP*_x_*//Ni@RGO/CF	–	33	8.97	3510	5000 (93.7%)	1.6	[[qv: 42c]]

“–” means that the data are not available. *C*_A_ and *C*_V_ are the areal capacitance and volumetric capacitance, respectively. *E*_V_ is the maximum volumetric energy density. *P*_V_ is the maximum volumetric power density.

The potential use of our device in flexible/portable electronics has been preliminarily evidenced by the electrochemical testing under bending states. As shown in Figure 4f, the device can be readily bent with different degrees. Both the CVs and electrochemical impedance spectroscopy (EIS) data reveal negligible changes upon bending the device from 0° to 360° and then back to 0° (inset in Figure 4f). While twisted, it can power one red light‐emitting diode (LED) efficiently, as illustrated in the inset of Figure 4e. Such an excellent performance durability should be due to the high mechanical flexibility of the wire electrodes together with the integration with the quasi‐solid‐state PVA–LiCl electrolyte. The electrolyte solidifies during the device assembly and acts like a glue that holds the two electrodes together, ensuring the structural integrity even being subjected to extreme shape deformation.

### 2.4 Device Scale‐Up and Potential Application Demonstration

**Figure 5:**
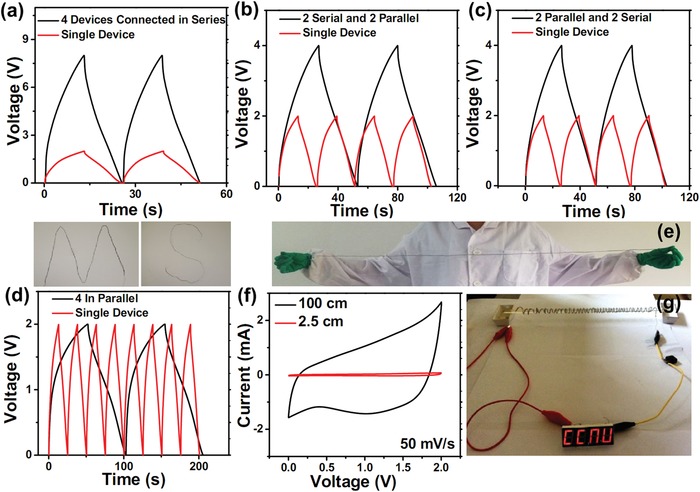
Galvanostatic charge–discharge curves for four WQAPs connected a) in series, b,c) in a combination of series and parallel, and d) in parallel. e) A long WQAP with a length of 100 cm, which can be facilely designed with different shapes. f) CV curve comparison of the WQAP with lengths of 2.5 and 100 cm. g) The CCNU LED pattern lighted up by the 100 cm WQAP device.

For a real application, portable electronic devices often require cells to be packaged in series, in parallel, or in combinations in order to meet the energy and power demands. Thus, it would be of great interest if wire‐shaped SCs could be controlled on the output voltage and current by using tandem serial and parallel assemblies with minimal energy losses. We have evaluated the charge–discharge performance by assembling four WQAPs in different configurations. As shown in **Figure** **5**a, compared with a single WQAP that operates at 2.0 V, the four WQAPs connected in series exhibit an 8.0 V voltage window with a similar discharging time. When the four WQAPs are combined two in parallel and two in series (combine “two that are in parallel” with “other two that are also in parallel” in series) or two in series and two in parallel (combine “two that are in series” with “other two that are in series” in parallel), both the output voltage and the discharging time are increased twice at the same charge–discharge current (Figure 5b,c). Assembling the four WQAPs in parallel, the output voltage is unchanged while the discharging time is four times that of a single cell when operating at the same current (Figure 5d). All the above results accord well with the laws of physics. In addition, the four combined devices show good triangular charge–discharge curves with a negligible *IR* drop, which is again indicative of excellent capacitive behaviors with minimal internal resistance.

The WQAP device fabrication can also be easily scaled up, and a long WQAP with a length of 100 cm is shown in Figure 5e. The device is also highly flexible, and could be bent into different shapes like M and S. With the scaling up, the capacitive behavior of the WQAP is still good, as evidenced by the quasi‐rectangular CV shape and 40 times increase of the CV integrated area (capacitance) as compared to the 2.5 cm WQAP (Figure 5f). As an application potential demonstration, the charged 100 cm WQAP surrounded on a glass rod can power a CCNU LED pattern brightly (Figure 5g).

## 3 Conclusions

In sum, hierarchical biphase core–shell nano‐MnO_2_ array cathode and MoO_2_@C nanofilm anode have been fabricated on tiny Ti wires and further utilized to assemble a novel WQAP device. The direct growth of electrode architectures on 1D current collector gives rise to excellent electron transport and ion diffusion efficiency, good flexibility, and solid interfacial contact with PVA–LiCl quasi‐solid‐state electrolyte. With the integration of the gel electrolyte, a 2.0 V WQAP device is developed, which manifests high volumetric energy and power densities (9.53 mWh cm^−3^ and 22 720 mW cm^−3^, respectively), and outstanding cycling stability up to 100 000 times. The overall electrochemical performance is better than those of previously reported WQAP devices and even comparable to those of some commercial devices. Beneficial to the facile synthetic techniques, our WQAP can be easily scaled up to 1 m, which still demonstrates good electrochemical attributes and can power LED patterns.

## 4 Experimental Section

*Synthesis of Hierarchical α‐MnO_2_ Nanorod@δ‐MnO_2_ Nanosheet Cathode*: First, 0.45 g KMnO_4_ and 0.065 mL HCl were added into a 30 mL aqueous solution containing 26 mL acetone and 4 mL deionized water under vigorous stirring at room temperature. Afterward, the mixed solution was transferred into the Teflon‐lined stainless steel autoclave with Ti wires (0.05 mm in diameter). The autoclave was then sealed and maintained at 200 °C for 9 h. After the hydrothermal growth, the Ti wires were taken out, washed with deionized water, and then dried in air. The obtained α‐MnO_2_ wire electrode was further immersed into a 60 mL aqueous solution containing 0.25 g glucose for 24 h. Subsequently, the wire was dried at 60 °C and further annealed at 450 °C in the flow of Ar for 2 h, which led to the coating of an amorphous carbon layer as a reactive template on the nanorod surface^7^ to grow δ‐MnO_2_ nanosheets by the facile interfacial reaction between KMnO_4_ and the carbon layer. In detail, a 60 mL solution containing 0.2844 g KMnO_4_ was heated in autoclave at 160 °C with the above α‐MnO_2_ nanorod–grown Ti wire immersed in. After 3 h, the Ti wire was taken out, washed with deionized water, and annealed at 450 °C for 40 min to improve the adhesion between MnO_2_ and Ti.

*Preparation of MoO_2_@C Nanofilm Anode*: The MoO_2_@C nanofilm anode was synthesized via a simple electrodeposition. The electrodeposition experiment was carried out at room temperature using an electrochemical workstation (CHI 760C, CH Instruments Inc., Shanghai) in a three‐electrode cell configuration. Ti wire was used as the working electrode, a Pt plate as the counter electrode, and a Ag/AgCl as the reference electrode. The deposition was performed in 0.05 m (NH_4_)_6_Mo_7_O_24_ and 0.03 m C_6_H_12_O_6_ aqueous electrolyte by cycling from 0 to −1.0 V with a sweep rate of 20 mV s^−1^ for 32 cycles. The as‐prepared sample on Ti wire was further annealed at 550 °C for 1 h in an Ar gas.

*Assembly of WQAP Device*: The wire WQAP was assembled with gel electrolyte of PVA–LiCl. First, the PVA–LiCl sol electrolyte was prepared as follows. 8 g PVA powder, 6 g LiCl, and 50 mL deionized water were mixed and heated to 85 °C with stirring until the solution became clear. Then, both the cathode and anode wires were painted with PVA–LiCl sol electrolyte, and with partial gelation the wires were further twisted together to finally form a PVA–LiCl gel integrated WQAP.

*Characterizations*: The wire electrodes and WQAP device were characterized by using SEM (JSM‐6700F), TEM (JEM‐2010FEF; 200 kV) with EDX analysis, Raman spectroscopy (Witech CRM200; 532 nm), and XRD (Bruker D‐8 Avance).

*Electrochemical Tests of Individual Electrodes*: A three‐electrode mode consisting of a Ag/AgCl as the reference electrode, a platinum plate as the counter electrode, and the MnO_2_‐grown wire or MoO_2_@C‐grown wire as the working electrode in 2 m LiCl electrolyte was utilized to investigate the charge storage properties of individual electrodes. EIS data were measured at frequencies ranging from 0.01 to 100 kHz with a potential amplitude of 5 mV.

The areal capacitance (*C*_A_) was calculated according to the following equation: *C*_A_ = *C*_Electrode_/*A*_wire_, where *C*_Electrode_ is the measured capacitance and *A*_wire_ is the surface area of the wire.

The volumetric capacitance (*C*_V_) was calculated by the following equation: *C*_V_ = *C*_Electrode_/*V*_wire_ = *C*_Electrode_/(*πr^2^L*_wire_), where *L*_wire_ and *r* are the length and radius of the wire, respectively.

*C*_Electrode_ was calculated from the galvanostatic discharge curve using the following equation: *C*_Electrode_ = *It*/*U*, where *I* is the discharge current, *t* is the discharge time, and *U* is the potential window excluding the *IR* drop.

*Electrochemical Measurements of Full Cells*: The capacitance of the full cells was measured in a two‐electrode mode based on galvanostatic charge/discharge curves. Hierarchical MnO_2_ and MoO_2_@C‐based wires were employed as the positive and negative electrodes, respectively. 2 m LiCl or PVA–LiCl gel was used as the electrolyte. Specifically, *C*_cell_ = *It*/*U*_cell_, where *U*_cell_ is the voltage window excluding the *IR* drop. The device's areal and volumetric capacitances were calculated based on the following equations: *C*_cell,A_ = *C*_cell_/*A*_cell_ and *C*_cell,V_ = *C*_cell_/*V*_cell_, where *A*_cell_ is the surface area of one wire electrode and *V*_cell_ refers to the device volume. *V*_cell_ was obtained by considering the volumes of two electrolyte‐coated electrode wires (the diameter of the electrode wires after electrolyte solidification was measured based on the inset in Figure 4a).

The volumetric energy density was obtained from the following equation: *E*_cell,V_ = ½*C*_cell,V_ × *U*_cell_^2^, and the volumetric power density was estimated based on the following equation: *P*_cell,V_ = *E*_cell,V_/*t*_discharge_, where *t*_discharge_ is the discharge time of the device.

## Conflict of Interest

The authors declare no conflict of interest.

## Supporting information

Supplementary
