# Supplementary material for: Scalable Wire‐Type Asymmetric Pseudocapacitor Achieving High Volumetric Energy/Power Densities and Ultralong Cycling Stability of 100 000 Times
Source: Adv Sci (Weinh). 2019 Mar 7;6(10):1802067. doi: 10.1002/advs.201802067 (PMC6524125; doi:10.1002/advs.201802067)
Supplement: Supplementary file 1 — Supplementary [file ADVS-6-1802067-s001.pdf]

Copyright WILEY-VCH Verlag GmbH & Co. KGaA, 69469 Weinheim, Germany, 2019.

ADVANCED SCIENCE
Open Access

Supporting Information

for *Adv. Sci.,* DOI: 10.1002/advs.201802067

Scalable Wire-Type Asymmetric Pseudocapacitor Achieving High Volumetric Energy/Power Densities and Ultralong Cycling Stability of 100 000 Times

*Qiuyue Gui, Lingxia Wu, Yuanyuan Li, and Jinping Liu**

Copyright WILEY-VCH Verlag GmbH & Co. KGaA, 69469 Weinheim, Germany, 2019.

Supporting Information

For Adv. Sci., DOI: 10.1002/advs.201802067

Scalable Wire-Type Asymmetric Pseudocapacitor Achieving High Volumetric Energy/Power Densities and Ultralong Cycling Stability of 100,000 Times

Qiuyue Gui, Lingxia Wu, Yuanyuan Li and Jinping Liu*

Q.Y. Gui, Prof. J. P. Liu
School of Chemistry, Chemical Engineering and Life Science and
State Key Laboratory of Advanced Technology for Materials Synthesis and Processing
Wuhan University of Technology
Wuhan, Hubei 430070, P. R. China
E-mail: liujp@whut.edu.cn

L. X. Wu, Prof. J. P. Liu
Institute of Nanoscience and Nanotechnology
Department of Physics
Central China Normal University
Wuhan, Hubei 430079, P. R. China

Prof. Y. Y. Li
School of Optical and Electronic Information
Huazhong University of Science and Technology
Wuhan 430074, P. R. China

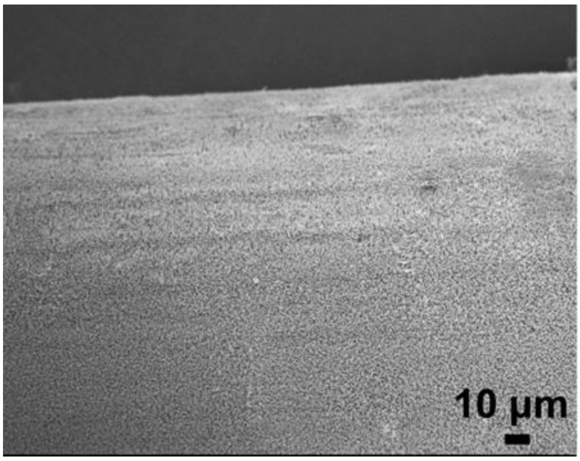

Figure S1. Low−resolution SEM image of α-MnO_2 nanorod array grown on Ti wire.

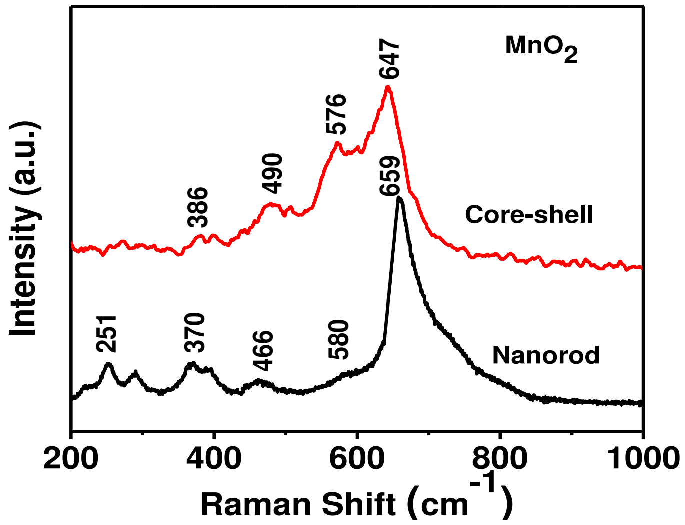

Figure S2. Raman spectra of α-MnO_2 nanorod array and hierarchical MnO_2 core-shell nanoarray.

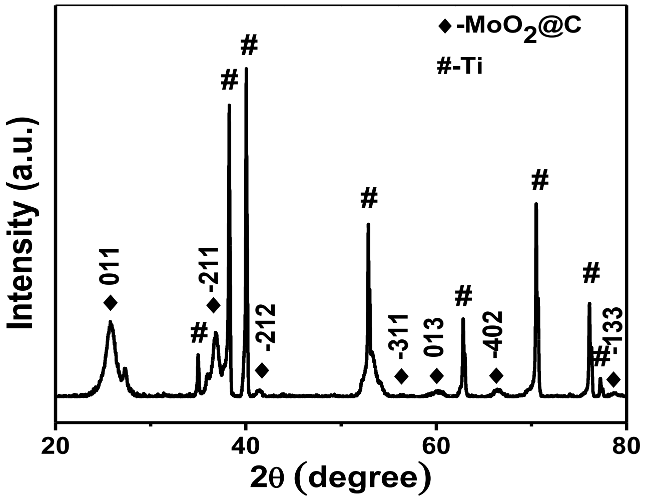

Figure S3. XRD pattern of MoO_2@C nanofilm.

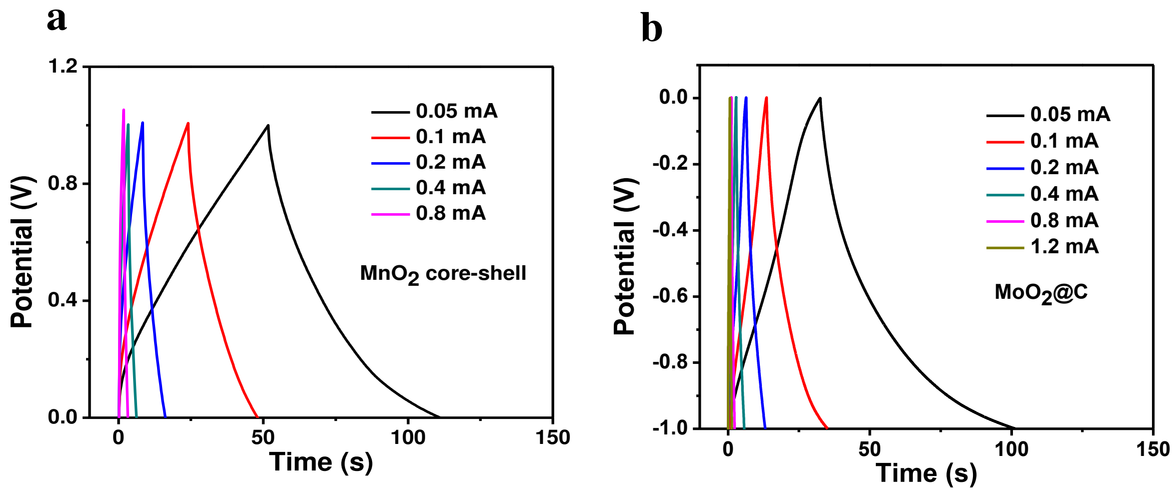

Figure S4. Galvanostatic charge-discharge profiles of the wire cathode and anode.

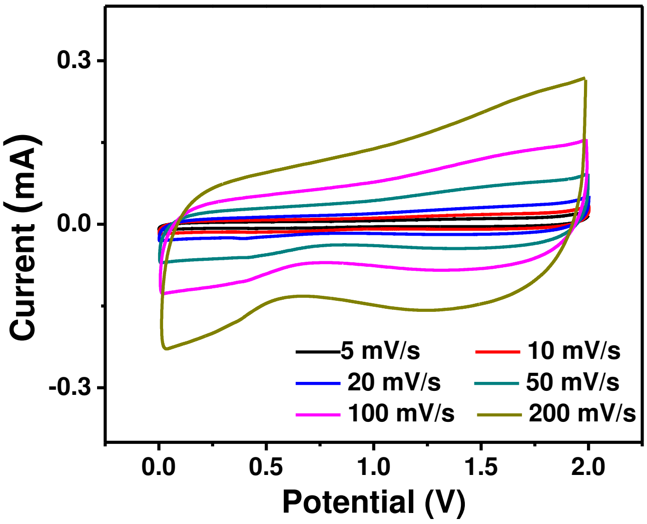

Figure S5. CV curves of the full-cell device in aqueous 2 M LiCl electrolyte.

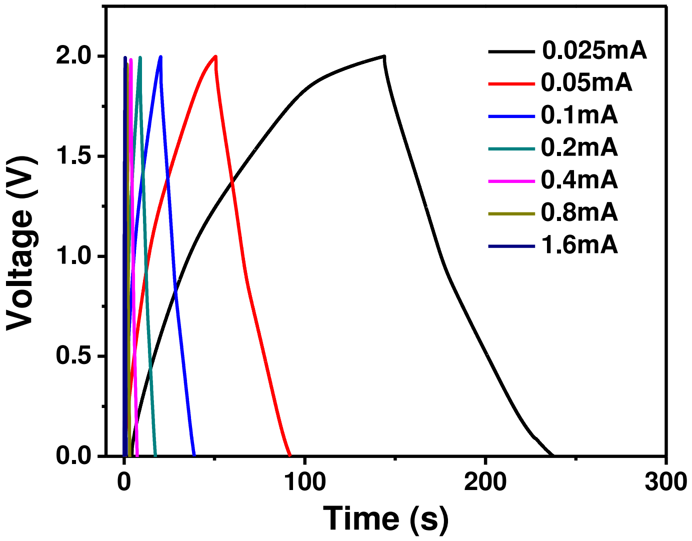

Figure S6. Charge-discharge curves of the full-cell device in aqueous 2 M LiCl electrolyte.

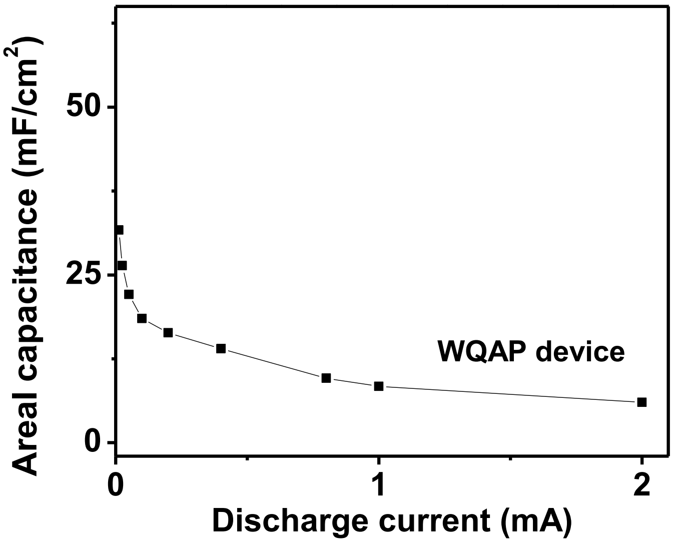

Figure S7. Rate capability of the WQAP device (α-MnO_2@δ-MnO_2//MoO_2@C) based on areal capacitance.
